# Minimal second language exposure, SES, and early word comprehension: New evidence from a direct assessment*

**DOI:** 10.1017/S1366728914000820

**Published:** 2016-01-22

**Authors:** Stephanie Deanda, Natalia Arias-Trejo, Diane Poulin-Dubois, Pascal Zesiger, Margaret Friend

**Affiliations:** San Diego State University, University of California, San Diego; Universidad Nacional Autonoma de Mexico; Concordia University; University of Geneva; San Diego State University

**Keywords:** Vocabulary, language exposure, socioeconomic status, English, Spanish

## Abstract

Although the extant literature provides robust evidence of the influence of language exposure and socioeconomic status (SES) on language acquisition, it is unknown how sensitive the early receptive vocabulary system is to these factors. The current study investigates effects of minimal second language exposure and SES on the comprehension vocabulary of 16-month-old children in the language in which they receive the greatest exposure. Study 1 revealed minimal second language exposure and SES exert significant and independent effects on a direct measure of vocabulary comprehension in English-dominant and English monolingual children (N = 72). In Study 2, we replicated the effect of minimal second language exposure in Spanish-dominant and Spanish monolingual children (N = 86), however no effect of SES on vocabulary was obtained. Our results emphasize the sensitivity of the language system to minimal changes in the environment in early development.

The number of Americans who speak a language other than English has risen to one in every five residents, such that children in the U.S. have become increasingly exposed to languages other than English (U.S. Census Bureau, 2011a). Additionally, it is often the case that these children belong to a lower socioeconomic status (SES) relative to children exposed exclusively to English (U.S. Census Bureau, 2011b). Our understanding of both the processes and outcomes of language acquisition in this population is limited in a number of ways. For example, the majority of extant studies in this area focus on language production. Thus little is known about the processes of early acquisition, specifically comprehension, and how these transition into production among children learning more than one language. Of interest then, is whether (and how early) the factors that are known to influence language production also influence comprehension.

Two factors known to be strong predictors of language outcomes in young children are relative language exposure and SES (Hoff, 2013). There is a dearth of evidence on the influence of these factors on language development as early as the second year of life. This is of particular importance since this is a period of rapid growth in the language system and, as such, may be especially sensitive to even small perturbations in the language environment. Of central importance to the present study are the effects of minimal second language exposure on the dominant language. Is 80% exposure to a language different from 100%? From a clinical perspective, it is of practical interest to understand whether a minimal reduction in language exposure slows acquisition in the dominant language (i.e., for our purposes, the language to which children receive the greatest exposure), or whether children with minimal second language exposure develop at rates comparable to their monolingual peers. Taken together, it is crucial for researchers and clinicians alike to understand the impact of SES and minimal second language exposure at early stages of language acquisition to better understand development in this growing population.

Several factors limit our understanding of the effects of exposure and SES on early language acquisition. First, studies frequently use parent report such as the MacArthur Communicative Development Inventories (MCDI; Fenson, Marchman, Thal, Dale, Reznick & Bates, 2006) to estimate the size of children’s vocabulary. Although parent reports are useful in providing a broad inventory of productive vocabulary, the indirect nature of parent report is a concern when estimating comprehension vocabulary since parents have less evidence, relative to production, of their child’s word knowledge (Tomasello & Mervis, 1994; Stiles, 1994). Consequently, replication with direct assessment is important. Second, as mentioned previously, most extant work investigating effects of SES and relative language exposure has focused on language production (Hart & Risley, 1995; Hoff & Tian, 2005; Hoff, 2003). A key question then is whether similar effects obtain earlier in comprehension. Indeed the majority of research on SES, relative language exposure, and vocabulary has been conducted on school-aged children. Whether there are effects of SES and relative language exposure in children well before this age and whether these effects obtain cross-linguistically is unknown. Finally, it is not known whether minimal exposure to a second language influences the course of acquisition in the dominant language. Given the relative immaturity of the lexical system prior to the onset of word production, one possibility is that it may be particularly sensitive to the effects of both minimal second language exposure and SES.

To address these limitations, the current work assesses early comprehension and production using parent report and supplements this assessment with a direct measure of comprehension. Further, we contrast two language groups, English-learning and Spanish-learning children, to explore the influence of SES and minimal exposure in an understudied sample. In what follows, we first review literature on relative language exposure and language acquisition, then turn to work on SES and early vocabulary development, and put forward a set of predictions.

## Language exposure

Research examining variability in lexical knowledge in children exposed to more than one language shows a significant positive relation between relative language exposure and expressive vocabulary (Pearson, Fernandez, Lewedeg & Oller, 1997; Eilers, Pearson, and Cobo-Lewis, 2006; David & Wei, 2008; Poulin-Dubois, Bialystok, Blaye, Polonia & Yott, 2013; Bedore, Peña, Summers, Boerger, Resendiz, Greene, Bohman & Gillam, 2012). Relative language exposure in young bilinguals is typically measured via parent report. Generally, parents are asked how many hours of each language the child hears, and the researcher then calculates proportions of exposure to each language. Typically, parent reports are collected through an interview, a checklist, or a daily diary (e.g., Bedore et al., 2012; Bosch & Sebastian-Galles, 1997; Marchman, Martínez-Sussmann & Dale, 2004; Parra, Hoff & Core, 2011; Thordardottir, Rothenberg, Rivard & Naves, 2006). These calculations provide an estimate of relative exposure in a child who hears more than one language and can aid in classifying a child as monolingual or bi/multi-lingual. For our purposes, a child with 80 percent exposure to English, and 20 percent exposure to Spanish was considered dominant in English. A child with 100 percent exposure to English was classified as an English monolingual. However, there are many definitions of language dominance beyond those based on language exposure. These are typically measures of productive proficiency such as MLU, total number of utterances, and word types (e.g., Genesee, Nicoladis & Paradis, 1995; Kupisch, 2007). For the purposes of this paper, the terms English- and Spanish-dominant, and English and Spanish monolingual reflect differences in exposure but not necessarily proficiency, in each language.

Although a relation between relative language exposure and productive vocabulary is well documented, subsequent research has provided evidence that this relation is not linear. In a study using direct behavioral assessments of five-year-old children learning French and English, Thordardottir (2011) found that children who had 35 percent exposure to a language had comprehension vocabularies that did not differ significantly from monolinguals. This suggests a relatively low exposure threshold for monolingual competency at 5 years of age. However, Thordardottir’s findings were based on a highly educated Canadian sample and reports of exposure over the child’s five years of life were retrospective, therefore limiting the extension of these findings. Even so, similar results have been found in five-year-old children, of less educated mothers, exposed to Spanish and English in the U.S. using language experience composite scores from reports of current language input and child output (Gibson, Peña & Bedore, 2012). Together, results from studies reflect the influence of exposure on vocabulary proficiency near the time of school entry when, normatively, the oral language system is relatively well established. As a consequence, these findings tell us little about the influence of exposure much earlier in development. That is, it is not known whether second language exposure, even at minimal levels, exerts an influence early in development when the language system is just emerging.

The current study assesses vocabulary knowledge in children using a direct measure of comprehension before the onset of production. In this way, we investigate the effects of minimal second language exposure on vocabulary acquisition in the dominant language. We focus on minimal changes in exposure to test the limits of the emerging language system with regard to its sensitivity to early language input. Although extant studies include children who experience minimal second language exposure, no study to date has directly assessed the relationship between minimal exposure and vocabulary knowledge in children prior to school entry. However, there is evidence suggesting that minimal exposure influences acquisition. For example, five-year-olds with at least 80% exposure to the dominant language reveal second language knowledge of morphosyntax and semantics despite limited (< 20%) second language exposure (Bedore et al., 2012). Similarly, Spanish–English bilinguals between 8 and 30 months of age have been reported to produce words in the less-dominant language on the MCDI (< 20% exposure to the second language, Pearson et al., 1997). These findings speak to the effects of minimal exposure on second language acquisition, but how minimal exposure to a second language affects vocabulary comprehension in the dominant language prior to age 2 is unknown. We now turn our attention to a second factor that has been shown to influence vocabulary size in young children: SES.

## Socioeconomic status

Mounting research documents positive relations between the quantity of language input, SES, and lexical development in children. In seminal work documenting these relations, Hart and Risley (1995) obtained monthly home language samples when children were between 10 and 36 months of age. Based on these language sample data, Hart and Risley used the number of word tokens to estimate both total parent language input and the trajectory of child language production. They found differences in both the quality and quantity of parent language input as a function of SES, which consequently, correlated with vocabulary size in young children. Additionally, differences were maintained longitudinally, such that children from higher SES homes developed larger vocabularies by 3 years of age as compared to those from lower SES homes. Notably, the effect of SES was apparent at approximately two years of age, when their expressive vocabularies were emerging. The early emergence of this difference, in part, motivates the present research to explore the effects of SES on comprehension prior to significant growth in production.

Unlike the early work by Hart and Risley, the research on vocabulary development in the early preschool years has relied largely on parent-report measures of lexical knowledge. Parent reports are useful in that they provide a representative measure of language, as parents observe the child on a daily basis and across a variety of contexts. Additionally, parent report assessments are a relatively inexpensive way to collect a large inventory of child language measures. These measures are also not affected by child temperament, unlike more direct methods. They are limited, however, in that they assess early language indirectly, and are not devoid of potential reporter biases.

One widely used parent-report measure is the MCDI (Fenson et al., 2006). Results similar to those by Hart and Risley (1995) have been obtained using MCDI estimates of production. Specifically, in a study of two-year-old children, Arriaga, Fenton, Cronan and Pethick (1998) documented significantly lower expressive vocabulary percentile scores for low-income children relative to the middle-income MCDI norming sample (Fenson, Dale, Reznick, Bates, Thal, Pethick, Tamasello, Mervis & Stiles, 1994). That is, children from higher SES households were reported to say more words than those from lower SES households. Feldman, Dollaghan, Campbell, Kurs-Lasky, Janosky and Paradis (2000) replicated Arriaga et al.’s findings with regard to SES and vocabulary production in two-year-old children. Conversely however, at one year of age, Feldman et al. found that lower SES parents reported greater language proficiency than higher SES parents in the number of phrases understood, words comprehended, and words produced.

Feldman et al. posited that lower SES parents might systematically over-estimate child language. In response, Fenson, Bates, Dale, Goodman, Reznick and Thal (2003) pointed to the fact that early vocabulary is highly variable and noted that the combined findings across studies do not rule out the possibility of over- and under-estimations. Further, they contended that Feldman et al.’s observation of a negative relation between SES and vocabulary in young infants might result from the added difficulty of estimating knowledge in newly emerging language systems. Consistent with this idea, more recent research has revealed comparable accuracy in reporting on vocabulary production across levels of SES on the MCDI by comparing parent reports and child speech samples (Furey, 2011; Sachse & Suchodoletz, 2008). However, most studies with findings of over estimations mediated by SES are on reports of very early child comprehension on the MCDI (Reznick, 1990; Fenson et al., 1994; Feldman et al., 2000).

The current state of the literature thus calls for a direct assessment of early comprehension particularly for the purpose of assessing the effects of SES on acquisition. A relation between SES and language acquisition has been documented in direct assessments of vocabulary production in two-year-olds (Hart & Risley 1995; Hoff, 2003). Of interest is whether these effects obtain in the earlier-emerging vocabulary comprehension system when assessed directly. Although direct assessments of vocabulary comprehension exist and are widely used, such as the Peabody Picture Vocabulary Test (PPVT, Dunn & Dunn, 2007), they are not appropriate for children less than 30 months of age. Consequently, the effects of SES on vocabulary comprehension prior to age 2 are not well understood.

It is important to note that direct measures are not necessarily ‘better’ than indirect, parent-report measures. Rather we think of them as complementary. For example, parent report may provide the better estimate of the sort of contextualized vocabulary knowledge seen in the home where the child is surrounded by familiar objects that may serve as cues to the association between words and referents. Conversely, direct measures of vocabulary comprehension, in which a child is asked to “point to the shoe” for example, may provide the better estimate of strong, decontextualized word representations which allow a child to correctly identify a referent in the absence of familiar, supportive cues to word meaning. Nevertheless, indirect measures are subject to reporting effects that make clarifying the role of SES in early language difficult. Thus, one purpose of this paper is to ask whether SES effects are observed when vocabulary is assessed directly.

## Study aims and predictions

The first aim of the current study is to assess whether minimal second language exposure (≤ 20%) influences language development in the second year of life. That is, do children with 100% versus 80% exposure to their first language demonstrate comparable lexical development? Recall Thordardottir’s (2011) finding that 35 percent exposure was necessary to reach levels of receptive vocabulary comparable to monolinguals. Extrapolating from these findings to minimal second language exposure in younger children, one might not expect to see effects on vocabulary comprehension in the dominant language. Alternatively, it is possible that the vocabulary system is particularly sensitive at early ages, such that even minimal exposure to a second language is influential when vocabulary comprehension is newly emerging. Following this reasoning, we expected a significant effect of minimal second language exposure on word comprehension in the dominant language at 16 months of age. Oller and Eilers (2002) provide some evidence for this hypothesis. In a cross-sectional study of bilingual children in kindergarten, second, and fifth grades, the effects of exposure on child vocabulary performance vocabulary (both comprehension and production) were larger at younger ages and waned over time. That is, bilingual children eventually reached monolingual norms in their dominant language even when their exposure was split between two languages. Similarly, in a study with children in Head-Start programs, effects of exposure narrowed over a two-year period (Hammer, Lawrence & Miccio, 2008). In the present paper, we contrast children who receive 100% exposure to a single language with those who receive 80% exposure and investigate the influence of minimal exposure to a second language on comprehension and production in the dominant language at 16 months of age. Further, we assess the effects of minimal exposure in English-speaking children and extend this research to an understudied sample of Spanish-speaking children. Based on the notion that the effects of exposure may be amplified early in acquisition we expected minimal exposure to a second language to exert an effect on vocabulary in the dominant language.

The second aim is to investigate the effects of SES on vocabulary comprehension in 16 month-old-children using a direct child performance measure and to contrast this with parent reported comprehension and production on the MCDI. Extant studies on the effects SES on language acquisition largely focus on vocabulary production. The one recent study to assess the effects of SES using both parent report on the MCDI and a direct measure of early comprehension found that children from lower-SES households were reported to know fewer words and were less efficient in processing words relative to their higher-SES peers at 18 months (Fernald, Marchman & Weisleder, 2013). In the present paper, we assess comprehension and production using parent report and supplement this with a direct assessment of comprehension to clarify the influence of SES on acquisition at 16 months of age. In light of the recent work by Fernald et al., (2013) and given that comprehension precedes and is linked to production (Benedict, 1979; Hoff, 2001), we anticipate that vocabulary comprehension in young children will be positively and significantly influenced by SES.

In sum, the extant literature on language exposure and vocabulary is such that it focuses largely on vocabulary production and on school-aged children with relatively balanced exposure across two languages. The current state of research on SES and early vocabulary is such that the majority of findings come from language production or parent reported comprehension. To date, the effects of minimal language exposure and SES on vocabulary have not been examined conjointly in a single sample. The following questions guide the present research:

1. Do SES and exposure effects previously documented in language production extend to early comprehension?
2. Specifically, does minimal exposure to a second language influence vocabulary acquisition in the dominant language?
3. Are effects of SES present in the vocabulary acquisition of children exposed exclusively to one versus two languages?

## Study 1

### Method

#### Participants

Participants were recruited through birth records, flyer postings, and child-oriented events in a large metropolitan area in the U.S. These participants formed part of a larger longitudinal study aimed at documenting acquisition in the dominant language and its implications for subsequent development. All participants had normal hearing and vision.

Seventy-two English-dominant and English monolingual children (*M* = 16;21 months, range = 15;15 – 18;0) participated in Study 1. At least 80 percent of the language they heard was English. The average maternal education was 15.4 years (some college, range = 12–18 years). See Tables 1 and 2 for the demographic and language exposure characteristics of this sample.

#### Measures

The Language Exposure Questionnaire (LEQ; Bosch & Sebastian-Galles, 1997) acquires parent reports on quantitative and qualitative aspects of language exposure and there is evidence supporting the validity of such reports (Goodz, 1989; Parra et al., 2011). For each language, parents were interviewed by a trained experimenter about the number of speakers who interacted with the child and the number of hours of exposure to each speaker over the course of the child’s life. To be included in the questionnaire, speakers must have interacted with the child at least once per week. Relative language exposure was estimated by calculating the proportion of time that the child heard English relative to other language input.

The interview was conducted over the phone prior to the child’s visit and lasted about ten to fifteen minutes. The LEQ was an electronic adaptation from an earlier hard-copy version developed by Bosch and Sebastian-Galles (1997). Data were entered into an excel file to enable quick calculations of language exposure. The interviewers administering the questionnaire were fluent speakers of English and Spanish and were trained to follow a detailed protocol outlining specific questions to be asked to elicit responses for the LEQ. For the English-dominant and English monolingual sample, the LEQ was administered with the primary caregiver in English.

The Computerized Comprehension Task contains 41 pairs of images presented on a touch sensitive screen (Friend & Keplinger, 2003; Friend & Keplinger, 2008; Friend, Schmidt & Simpson, 2012). Infants are prompted to touch the target by an experimenter (“Where is the shoe? Touch shoe.”). The 41 pairs of images consist of a target and a distractor. A touch to the target image produces a reinforcing sound. These pairs of images provided two test forms, such that the targets from Form 1 were the distractors in Form 2. Forms were counterbalanced across participants. The image pairs represent nouns, verbs, and adjectives at varying levels of difficulty. Difficulty level was defined based on normative data for 16 month-old children (Dale & Fenson, 1996). Difficult words are comprehended by less than 33%, moderate words are comprehended by 33 to 66%, and easy words are comprehended by more 66% of children at 16 months of age. By this definition, a third of the items were easy, a third were moderately difficult, and a third were difficult. Due to this distribution of difficulty, children typically recognize between 25 and 30% of the items at 16 months of age. Word pairs within trials were matched on difficulty and word class. A list of the CCT test items by difficulty level is presented in Appendix A.

The CCT begins with 4 training trials with no time limit. If the child touches the screen at least once, whether to the target or the distractor, the child moves on to the test phase after the 4 training trials. However, any incorrect touch during the training trials is followed by a correct touch to the target by the experimenter to model the desired response. If no touch has been made after repeating the training phase a second time, the child does not continue on to the test phase. All children proceeded to the test phase.

During the test phase, the experimenter presents the pairs of images immediately following the first mention of the target word in the prompt. After seven seconds elapse, if no response has been made, the trial ends and the pair of images disappears. The CCT has shown significant immediate test-retest reliability, thus suggesting that performance is systematic, as well as convergent validity with MCDI reports of vocabulary comprehension and 4-month test-retest reliability (Friend & Keplinger, 2008; Friend & Zesiger, 2011). CCT data were only collected in the child’s dominant language of exposure given the very low exposure to a second language, the nature of the larger study, and the driving question of the present study: does minimal exposure to a second language influence early acquisition in the dominant language of exposure? Further, the structure of this task is not conducive to measuring word knowledge in children with very low language exposure.

The MCDI is a widely used parent report measure of early language. The Words and Gestures version of the inventory, intended for children between 8 and 18 months of age, is a 396-item checklist allowing parents to mark the words their child understands and says. The inventory provides researchers with an indirect account of the child’s vocabulary comprehension and production. The MCDI has strong psychometric properties, including significant internal consistency, test-retest reliability, and convergent validity (Fenson et al., 1994). MCDI data were collected only in the child’s dominant language of exposure for the reasons cited above.

#### Procedure

The LEQ was administered over the phone prior to the child’s visit to the lab. Once in the lab, participants spent approximately 10 minutes playing with the experimenter, to insure that they were comfortable with the environment and the experimenter. Children and their parents were then escorted to a dimly-lit room which housed the CCT. All children sat on the caregiver’s lap, centered and approximately 35 centimeters from the screen. Parents listened to music over sound-cancelling headphones and wore dark glasses with blacked-out lenses to prevent any cueing of the children. Following the CCT, parents completed the MCDI: Words and Gestures. Participants were instructed on completing the MCDI consistent with the guidelines provided by Fenson et al. (2006). The MCDI was completed in the lab to insure that it was completed in comparable conditions across participants.

#### Categorization of SES and exposure

Historically, a variety of measures have been used to operationalize SES in studies of early language acquisition (e.g., Hollingshead Index, Medicaid enrollment, school lunch program). The majority of these studies also include maternal education as an index. In the present study we use maternal education as a proxy for SES for several reasons. First, the relation between SES and language development is mediated by differences in maternal input, which are directly related to parental education levels across various cultures (Dollaghan, Campbell, Paradise, Feldman, Janosky, Pitcairn & Kurs-Lasky, 1999; Hoff, 2003; Hoff & Tian, 2005). That is, among children of high- and mid-SES families, the relation between SES and child vocabulary is no longer significant once maternal speech variables are removed (Hoff, 2003). Secondly, Friend, Schmitt and Simpson (2012) found maternal education was a significant, though modest, predictor of CCT scores such that children of college-educated mothers knew more words than those with less educated mothers. Finally, income data were not equally available across samples in Study 2, thus to facilitate comparison across studies, and following this previous work documenting its validity, maternal education was used as a proxy for SES.

To assess differences in comprehension and production as a function of SES, children were split into two groups (higher SES and lower SES) based upon whether their mothers were at or above the median maternal education level for the sample (16 years of education, equivalent to 4 years of college). This cut point makes sense practically as it demarcates mothers who have completed college versus those who have not. Further, this categorical definition is supported by research showing discrete patterns in early brain development associated with different levels of parental education (Noble, Houston, Kan & Sowell, 2012).

For the lower-SES group, mean maternal education was equivalent to approximately 1 year post high school (*N* = 29; *M* = 13.2, range = 12 – 15). For the higher-SES group, mean maternal education was equivalent to completion of college (*N* = 43; *M* = 16.9; years range = 16 – 18 years). Analyses revealed no significant difference in maternal education between English monolingual and English-dominant children.

For the purpose of identifying children with minimal second language exposure, we divided children into two groups based on whether they were exposed to a language other than English. One group of 47 children had exposure only to English (English monolingual; English exposure = 100%), whereas the second group of 25 children had exposure to at least one other language, but no greater than 20% (English-dominant; mean second language exposure = 7%, range = 1 – 19%, see Table 2 for the language exposure characteristics of this group). Analyses revealed no significant difference in English language exposure as a function of maternal education.

### Results

Table 3 provides descriptive statistics on all measures of vocabulary as a function of SES and language exposure group. Scores on the CCT ranged from 0 to 31 words correctly identified. Similarly, MCDI comprehension scores ranged from 52 to 396 words, utilizing the full range of the scale, and MCDI production scores ranged from 0 to 233 words. MCDI vocabulary comprehension and CCT scores were normally distributed. However, MCDI productive vocabulary reports were positively skewed, which is expected given that most children at this age produce few words. Analyses revealed significant correlations between the CCT and MCDI vocabulary comprehension (*r*(72) = .38, *p* = .001), the CCT and MCDI vocabulary production (*r*(72) = .35, *p* = .003), and the MCDI comprehension and production vocabulary reports (*r*(72) = .67, *p* < .001). In addition, the MCDI and both forms of the CCT showed excellent internal consistency (Cronbach’s α = .95, .91, and .95, respectively). Together, these data indicate shared variance between our direct and indirect measures of comprehension, as well as between comprehension and production. Children who had more words reported by parents also tended to correctly identify more words on the CCT. Further, both parents and children exhibited within-measure consistency in their responses.

An Omnibus Repeated Measures ANCOVA with CCT form (2), gender (2) as between-subjects factors, age as the covariate, and vocabulary (CCT comprehension, MCDI comprehension, and MCDI production) as the repeated dependent measure revealed no significant effect of the predictor variables or of the covariate. As a consequence these variables were dropped from further analyses.

A Repeated Measures Analyses of Variance (ANOVA) with SES (2) and Language Exposure (2) as between-subjects factors, and with Vocabulary (CCT comprehension, MCDI comprehension, MCDI production) as the repeated dependent measure revealed a significant main effect of Language Exposure (*F*(1, 68) = 7.69, *p* = .007, ŋ^2^_p_ = .1) and a significant interaction of SES and Vocabulary (*F*(1, 68) = 5.84, *p* = .02, ŋ^2^_p_ = .04). There was no significant three-way interaction between SES, Exposure, and Vocabulary: SES and Exposure exerted independent effects on vocabulary.

#### Language exposure

Follow-up comparisons were conducted using Bonferroni familywise α = .017 per test. On the CCT, English monolinguals correctly identified 34 percent of the words whereas English-dominant children identified 21 percent on average (*t*(70) = 2.71, *p* = .009, ŋ^2^ = .1). MCDI production revealed the same pattern, with reports of production in monolingual infants at 50 words compared to 23 words in English-dominant infants (*t*(55.5) = 3.13, *p* = .003, ŋ^2^ = .07). Similarly, parents of monolinguals reported comprehension of 196 words on the MCDI, compared to 156 words for English-dominant children but this difference was not significant at the Bonferroni-adjusted familywise alpha level (*t*(70) = 2.25, *p* = .03, ŋ^2^ = .07).

#### Socioeconomic status

Follow-up comparisons (familywise α = .017) revealed that the SES X Vocabulary interaction reflects significant SES differences in vocabulary comprehension on the CCT (*t*(70) = 2.5, *p* = .015, ŋ^2^ = .08). Higher SES children identified the referent for an average of 34 percent of the words on the CCT whereas lower SES children identified significantly fewer words (*M* = 23%). Similarly, on the MCDI, higher SES parents reported that their children produced approximately 49 words, whereas lower SES parents reported production of 27 words, but this difference was not significant at the adjusted alpha level (*t*(57.6) = 2.27, *p* = .027, ŋ^2^ = .05). There was no significant effect of SES on MCDI comprehension reports.

### Discussion

In sum, and in accordance with our predictions, both exposure and SES influenced children’s vocabulary comprehension in the dominant language on the CCT. MCDI production was significantly influenced by minimal second language exposure, but not by SES whereas MCDI comprehension did not reveal significant effects of either SES or exposure.

One limitation of this study, however, is that we report a new finding of early minimal second language exposure effects in one sample of English-dominant participants. Indeed, although there are many children in the U.S. who are exposed only to English, there are also many children who are only minimally exposed to English early in the course of language acquisition. In fact, a growing number of Spanish monolingual children in the U.S. encounter systematic English exposure only upon school entry. One recent estimate is that the population of children between the ages of 5 and 17 in the U.S. public school system who hear a language other than English at home has increased from 4 million to 11 million between 1980 and 2009 (Aud, Hussar, Planty, Snyder, Bianco, Fox, Frohlich, Kemp & Drake, 2010). To follow-up on our results in Study 1, we assess vocabulary in Spanish-dominant children in the U.S., as well as in Spanish monolinguals living in Mexico who are not exposed to English, to assess whether minimal second language exposure and SES effects obtain across cultural and linguistic settings. That is, do effects of SES and minimal exposure to a second language extend to vocabulary comprehension in Spanish speakers? We expected that findings from Study 1 would replicate in 16-month-old Spanish-dominant and Spanish monolingual children.

## Study 2

### Method

#### Participants: US sample

Fifty-two Spanish-speaking children were recruited to participate in this study (*M* = 16;27 months, range = 15;21 – 20;21, 27 boys, 25 girls) using the same recruitment methods described in Study 1. Sixteen children were reported to hear Spanish 100 percent of the time, whereas the other thirty-six children had no more than 20 percent exposure to English. The average maternal education for this sample was high school completion (*M* = 12.6, range = 6–18 years of education).

#### Participants: Mexico sample

Thirty-four Spanish monolingual children (100% exposure to Spanish) were also recruited to participate in the study (*M* = 17;0 months, range = 15;9 – 18;6, 22 boys, 12 girls). As in our other samples, all children had normal hearing and vision. Participants in the Mexico sample were recruited through flyer postings in a large metropolitan area. All children in this sample heard only Spanish. The average maternal education for this sample was 2 years of college (*M* = 14.7, range = 8–18 years of education).

#### Measures

The Spanish adaptations of the measures in Study 1 were used to assess the participants in Study 2. Specifically, the Spanish adaptation of the MCDI:WG (Inventario del Desarrollo de Habilidades Comunicativas, Primeras Palabras y Gestos, IDHC; Jackson-Maldonado, Thal, Fenson, Marchman, Newton & Conboy, 2003) was used to acquire parent report data and a Spanish adaptation of the CCT was administered (Friend & Keplinger, 2008) using culturally relevant words derived from the IDHC and corresponding to the same distribution of difficulty as the English CCT. A list of the Spanish CCT test items by difficulty level (as derived from the IDHC norms) is presented in Appendix B. Like the English CCT, the Spanish CCT shows significant immediate test-retest reliability, thus suggesting that performance is systematic. As in Study 1, the LEQ was administered to determine relative language exposure for each child. Those administering the LEQ were fluent in English and Spanish.

#### Procedure

The procedures in Study 2 were the same as in Study 1.

#### Preliminary analysis

We included SES and Exposure as covariates in an Omnibus ANCOVA with CCT Form (2), Gender (2), Age (covariate), and Testing Site (Mexico or U.S.) as between-subjects factors and Vocabulary (CCT, IDHC comprehension, and IDHC production) as the repeated measure. Results revealed no significant effect of CCT Form, Gender, Age, or Testing Site on Vocabulary and no interaction of Testing Site with SES or Exposure. These variables were dropped from subsequent analyses. Since Testing Site was not significantly related to SES or Exposure, the U.S. and Mexico samples were combined to exploit the full range of SES and minimal second language exposure (0 – 20%). See Tables 4 and 5 for the demographic and language exposure characteristics of these samples.

#### Categorization of SES and exposure

The procedure for categorization of SES and relative language exposure was the same as in Study 1, such that participants were grouped into high and low SES groups based on whether maternal education was at or above 4 years of college education. The average maternal education of the higher SES group was 16.6 years (*N* = 30, range = 16–18 years), and average maternal education of the lower SES group was 11.7 years (*N* = 57, range = 6– 15). Analyses revealed no significant difference in Spanish language exposure as a function of maternal education.

Similarly, children were divided into two groups based on exposure. One group of 50 children had exposure only to Spanish (Spanish monolingual; mean Spanish exposure = 100%), whereas the second group of 36 children had minimal exposure to at least one other language (Spanish-dominant; mean second language exposure = 13%, range = 1–20%). Analyses revealed no significant difference in maternal education between Spanish monolingual and Spanish-dominant children.

### Results

Table 6 provides descriptive statistics on all measures of vocabulary as a function of SES and language exposure group for participants in Study 2. Scores on the CCT ranged from 0 to 41 words correctly identified. A similarly variable range was found on the IDHC, comprehension vocabulary reports ranged from 17 to 403 words, utilizing the full range of the scale, and IDHC production scores ranged from 0 to 334. IDHC vocabulary comprehension and CCT scores were normally distributed. However, IDHC vocabulary production reports were positively skewed, which is expected given that most children at this age produce few words.

Analyses on the Mexican sample revealed a significant correlation between IDHC production and comprehension scores (*r*(35) = .5, *p* = .002). The CCT was correlated with both IDHC comprehension and production reports, but these were not significant consistent with previous research (Friend & Keplinger, 2008). This is different than what we found with the English sample. Recall that parent reports were correlated with child performance in that sample whereas, in the current sample, children for whom parents report a large number of early words are not necessarily the same children who are performing well on the CCT. Consistent with our findings for the English sample, however, the internal consistency on the IDHC and on both forms of the CCT was excellent (Cronbach’s α = .93, .94, and .90, respectively).

Similarly, there was a significant correlation between comprehension and production measures on the IDHC (*r*(52) = .56, *p* < .001), and only a marginal correlation between CCT comprehension scores and IDHC production reports (*r*(52) = .25, *p* = .07) for the U.S. Spanish-speaking sample. Internal consistency on the IDHC and on both forms of the CCT for the U.S. sample was high (Cronbach’s α = .96, .77, and .91, respectively).

A Repeated Measures ANOVA with SES (2) and Language Exposure (2) as between-subjects factors and Vocabulary (CCT comprehension, IDHC comprehension, IDHC production,) as the repeated measure revealed a significant main effect of Language Exposure (*F*(1, 82) = 5.99, *p* = .016, ŋ^2^_p_ = .068). There was no main effect of SES, and no significant three-way interaction between SES, Exposure, and Vocabulary.

It is possible that the absence of an SES effect in the Spanish sample was due to depressed maternal education relative to our English-dominant and English monolingual samples in Study 1. To explore this possibility, we first compared CCT scores from children from the top and bottom third of the SES distribution. This yielded a group of 30 children with mothers who completed four years of college, and a second group of 38 children with mothers who completed a high school diploma at most. A t-test revealed no significant difference in the proportion of words correctly identified on the CCT between these two groups. This also held for the other two measures of vocabulary (IDHC comprehension and production). In addition, applying a median-split procedure as in Study 1 for the high and low SES group (median for Study 2: 12 years of education) also did not yield SES effects. To determine whether SES effects were specific to a particular test site, we ran the same repeated measures ANOVA analyses for each site separately (Mexico vs. U.S.). Results revealed no effects of SES at either the U.S. or Mexico sites. Finally, we repeated the ANOVA with the annual family income, rather than maternal education, as the measure of SES in the U.S. sample^1^. Again, we found no significant effects of SES on any vocabulary measure (CCT comprehension, IDHC comprehension, IDHC production).

### Discussion

Similar to the English-speaking samples in Study 1, minimal second language exposure was related to Spanish vocabulary size on the CCT in Spanish-speaking children at 16 months of age. Conversely, SES was not related to vocabulary size, even when we compared the extreme ends of the SES spectrum, and regardless of whether we used maternal education or annual income as a proxy for SES. This is consistent with previous findings of differential effects of SES across English- and Spanish-speaking populations. For example, Hurtado, Fernald and Marchman (2008) found no relation of SES to vocabulary size and processing speed at 18 and 24 months of age in Spanish monolinguals, but did find relations among these variables in English monolinguals of the same age in a later study (Fernald, Marchman & Weisleder, 2013). Similar results have also been documented using the IDHC: Jackson-Maldonado, Thal, Marchman, Bates and Gutiérrez-Clellen (1993) found no relation between SES and vocabulary in children between 8 and 31 months of age. Given these convergent results, it is possible that effects of SES emerge at different times in English- and Spanish-speaking samples or that experiential effects on acquisition are not well captured by SES in Spanish speakers. However, it is currently unknown what mechanism accounts for these differences. For instance, it may be that maternal education does not function as a proxy for SES in the same way in Spanish and English samples. Alternatively it is important to remember that early language acquisition occurs in a cultural context. An interesting possibility is that the culture of parenting in Spanish-speaking families overrides effects of SES on early acquisition. We consider these possibilities in greater detail in the general discussion.

## General Discussion

### Minimal second language exposure and vocabulary in the dominant language

One aim of the current project was to determine whether vocabulary size in the dominant language is influenced by minimal exposure to a second language in the second year of life. Our results across English- and Spanish-speaking children revealed a significant difference in vocabulary size associated with minimal exposure to a second language such that children with 100% exposure to a language outperformed those with 80–99% exposure. The average exposure to a second language was 7% in Study 1 and 13% in Study 2. Our results are consistent with the “resource limitation hypothesis” which suggests that bilinguals face more challenges in word learning relative to monolinguals in using phonetic detail (Fennell, Byers-Heinlein & Werker, 2007; Fennell & Werker, 2003; Stager & Werker, 1997; Werker & Fennell, 2004; Werker, Fennell, Corcoran & Stager, 2002). Despite being exposed to greater phonetic breadth than monolinguals, bilinguals may have weaker phonemic representations by virtue of having relatively less exposure, since exposure is split across languages. Although bilinguals learn words at the same rate as monolinguals (Pearson, Fernandez & Oller, 1993), monolinguals seem utilize phonemic detail to guide word learning earlier.

These findings in conjunction with previous studies provide a developmental story that suggests that the effects of exposure narrow quickly over time. By age 20, 25, and 30 months, Hoff, Core, Place, Rumiche, Señor and Parra (2012) found no significant differences in English vocabulary size as measured on the MCDI between monolingual English and English-dominant (English exposure > 70%) children. Such an attenuation of exposure effects on vocabulary with age has been documented in school-aged children exposed to English and Spanish such that gaps in vocabulary narrowed over time (Hammer et al., 2008; Oller & Eilers, 2002). Indeed, by age 5, Thordardottir (2011) demonstrates that levels as low as 30–35% exposure are sufficient to reach monolingual vocabulary comprehension levels in French–English bilinguals. The pattern of results across studies suggests that although minimal exposure to a second language affects the vocabulary size of the dominant language at 16 months as shown in the present study, these effects might diminish, at least in production, as early as age 2 (Hoff et al., 2012). Further, extant research shows that early simultaneous bilinguals achieve native-like proficiency across syntax, semantics and vocabulary in the dominant language of the community as adults (Krashen, Scarcella & Long, 1982; Kupisch, 2012; Portocarrero, Burright & Donovick, 2007).

The present findings also speak to the effects of exposure across vocabulary production and comprehension. On the MCDI, vocabulary production and comprehension exhibited effects of minimal second language exposure, although effects on MCDI comprehension were less robust. Indeed, word comprehension may be harder to report than production increasing variability in reporting and limiting our ability to document effects of exposure. Even so, effects of minimal second language exposure on comprehension were evident using a direct assessment. Across our 16-month-old English- and Spanish-speakers, 80% exposure to a language was not sufficient to reach the vocabulary comprehension levels of their monolingual peers. It is important to note that there was wide variation in the second language to which children in our samples were exposed. It is possible that the effect of second language exposure on comprehension in the dominant language may be attenuated or exaggerated depending upon the specific languages to which the child is exposed. For example, bilinguals exhibit language transfer in vocabulary for highly related languages that share cognates (Perez, Mendez, Peña & Bedore, 2010). Thus, the effects of minimal second language exposure may vary with the level of similarity across the child’s languages.

Although the pattern of findings across this and other studies suggest an attenuation of exposure effects on vocabulary over time, future work must investigate these changes across comprehension and production in a single group of children. Our ongoing longitudinal research with this sample of children will help to determine whether effects of exposure will diminish with age, and whether these early differences in vocabulary have implications for later language outcomes.

### SES effects on vocabulary

A second aim of the current project was to assess the effects of SES on word knowledge using a direct measure of vocabulary comprehension and to contrast this with parent reported comprehension and production. Results from Study 1 revealed a relation between word knowledge and SES such that greater maternal educational attainment was associated with larger vocabularies in children. This pattern of results obtained for a direct measure of vocabulary comprehension (the CCT), thus replicating and extending prior research to the earlier-emerging vocabulary comprehension system (Hart & Risley 1995; Hoff, 2003; Hoff & Tian, 2005).

Conversely, SES was not significantly related to vocabulary size on the MCDI. This is in contrast to a paradoxical, negative, relation between reports of comprehension on the MCDI and SES previously reported in children before the second year of life (Feldman et al., 2000; Fenson et al., 1994; Reznick, 1990). As has been speculated by previous researchers, it is possible that a reporting bias relating to SES exists in parent reports of early vocabulary. Importantly, the current findings using a direct assessment reveal that vocabulary comprehension and SES are indeed positively related early in development for English-dominant and English monolingual children.

Results from Study 2, however, revealed that SES was not significantly related to vocabulary in Spanish-learning 16-month-olds. This is in contrast to a wealth of literature documenting SES effects in English-speaking children in the U.S. and in contrast to Study 1. Additionally, it has been shown that SES effects on language are mediated by maternal input, which is systematically related to maternal education, and therefore SES, in children within the second year of life (Hoff, 2003).

Despite previous work on maternal education and SES, one may argue that maternal education is not the best proxy for SES, and these results may not extend to Spanish-speaking children. However, at least two prior studies converge with our findings using different SES metrics and different measures of vocabulary. In one study, Hurtado et al. (2008) found no relations between the Hollingshead Index (a comprehensive measure of SES that includes maternal education) maternal input, and vocabulary size in 18- and 24-month-old Spanish-speaking children. This suggests that the influence of SES may emerge later, if at all, for Spanish-speaking to English-speaking children, as significant SES effects on vocabulary are found at both 18 and 24 months in English speakers (Hurtado et al., 2008; Fernald et al., 2013). In another set of studies, Jackson-Maldonado et al. (1993) found no significant relation between a number of SES variables (including maternal education) and rate of lexical development as measured on the IDHC in Spanish-speaking children between 8 and 31 months of age. Thus, our results converge with these studies showing no effect of SES on early vocabulary development in Spanish-speaking children using a range of vocabulary assessments and SES metrics. Indeed, our results also indicated no effects of SES when using family income rather than maternal education. Taken together, these results underscore the need for additional research on the growing Spanish-speaking demographic in the U.S. Although the current findings and those from other studies indicate differences among English- and Spanish-speaking populations, the mechanism for these disparities is currently unknown.

One possibility is that the differential effects of SES across the English (Study 1) and Spanish children (Study 2) might be tied to language input differences attributable to cultural practices. Although research suggests that SES effects are mediated by maternal input (Hart & Risley 1995; Hoff, 2003; Hoff & Tian, 2005), such a relation might not emerge across cultures. Consider, for example, that differences in maternal gesture and language are observed across ethnic groups even when controlling for maternal education (Tamis-LeMonda, Song, Leavell, Kahana-Kalman & Yoshikawa, 2012). Thus cultural differences in maternal input contribute variance that is unrelated to SES.

There is also evidence to suggest that parenting practices and knowledge of child development differ across cultures, and that this accounts for differences in language acquisition (Tamis-LeMonda & Kahana-Kalman, 2009; Rowe, 2008). Indeed, Mexican immigrant parents encourage obedience and collaboration more so than verbal communication and independence (Kayser & Guiberson, 2008; Greenfield, Trumbull, Keller, Rothstein-Fisch, Suzuki & Quiroz, 2006). As a consequence, we might expect to see variability in acquisition, particularly early in development, due to variability in maternal interaction style that is unrelated to SES but that has implications for language input.

### Limitations

A limitation of the current study is the lack of vocabulary assessment in the less-exposed language. CCT data collection for children with minimal second language exposure would have been challenging, as the potential for attrition is high when word knowledge is low (recall that the average exposure to a second language was only 7 and 13 percent for the Study 1 and Study 2, respectively). As such, our findings of reduced vocabulary attributable to SES and minimal second language exposure are limited to the dominant language. Previous work has shown, in fact, that total conceptual vocabulary summed across languages in bilinguals is comparable to vocabulary size in monolinguals at similar ages (Junker & Stockman, 2002; Marchman, Fernald & Hurtado 2010; Pearson et al., 1993; Pearson et al., 1997). However the primary goal of the present study was to assess the influence of minimal second language exposure on acquisition in the dominant language of exposure.

In addition, the CCT itself poses some possible limitations. For example, unlike parent-report measures such as the MCDI, the CCT is context-free and presents isolated two-dimensional images on a touch screen in the presence of a within-category distractor. Further, it requires a volitional response to indicate word knowledge. As such, it is likely that the CCT and the MCDI are differentially sensitive to robust word knowledge. That is, parent report measures likely tap into context-dependent, as well as decontextualized, word representations. Therefore, although parent report and direct vocabulary measures (like the CCT) are significantly correlated, each also captures unique vocabulary knowledge (Friend, in review). It is also possible to argue that the CCT does not measure word knowledge at all, but some extraneous characteristic (e.g., temperament) or cognitive ability (e.g., attention). However, recent work (Hendrickson & Friend, 2013; Hendrickson, Mitsven, Poulin-Dubois, Zesiger & Friend, in press) has shown that touch responses on the CCT converge with eye gaze and looking time patterns as measures of vocabulary proficiency.

### Summary and Conclusion

In sum, the current set of studies provides at least two important contributions. First, our findings confirm a relation between SES and early comprehension when assessed directly in English learning infants. This suggests that these effects may be masked in parent estimates in English-speaking children at 16 months of age. This finding clarifies previous research on SES effects on parent report. However, no effect of SES was apparent in the Spanish sample. Second, scores on the CCT reveal significant differences in vocabulary comprehension in the dominant language as a function of minimal second language exposure at 16 months of age in both English-learning and Spanish-learning children. By assessing comprehension directly, we extend existing research on language production to the earlier-emerging comprehension system. Further, we replicate the effects of minimal exposure to a second language in two separate samples and languages. Our results reveal the need to assess language acquisition across languages and cultures, as these findings demonstrate the sensitivity of the language system to minimal changes in the environment at the earliest stages of development.

## Figures and Tables

**Table 1:** Distribution of Selected Demographic Characteristics of Participants in Study 1 (age in months)

Characteristic	English monolingual mean age: 16;24 N = 47	English dominant mean age: 16;15 N = 25	Total N = 72
	Number (proportion) of participants		
Sex			
Female	23 (.49)	15 (.6)	38 (.53)
Male	24 (.51)	10 (.4)	34 (.47)
Maternal education			
High School	8 (.17)	3 (.12)	11 (.15)
Some College	8 (.17)	10 (.4)	18 (.25)
College Graduate	13 (.28)	9 (.36)	22 (.31)
Post-Baccalaureate	18 (.38)	3 (.12)	21 (.29)
Approximate income			
less than 34,000	5 (.11)	5 (.2)	10 (.14)
35,000 – 49,000	3 (.06)	2 (.08)	5 (.07)
50,000 – 74,000	7 (.15)	6 (.24)	13 (.18)
75,000 – 99,000	11 (.23)	7 (.28)	18 (.25)
100,000 – 150,000	13 (.28)	4 (.16)	17 (.24)
>150,000	7 (.15)	1 (.04)	8 (.11)
Declined to state	1 (.02)	0	1 (.01)
Ethnicity			
Asian	4 (.09)	3 (.12)	7 (.1)
Black/not Hispanic	2 (.04)	2 (.08)	4 (.06)
Hispanic	6 (.13)	10 (.4)	16 (.22)
White/not Hispanic	32 (.68)	7 (.28)	39 (.54)
Mixed Race	3 (.06)	3 (.12)	6 (.08)
Number of conversational speakers			
1–2	26 (.55)	8 (.32)	34 (.47)
3–4	16 (.34)	10 (.4)	26 (.36)
5–6	3 (.06)	6 (.24)	9 (.12)
7–8	2 (.05)	1 (.04)	3 (.04)
Outside-the-home care			
Yes	11 (.23)	8 (.32)	19 (.26)
No	36 (.77)	17 (.68)	53 (.74)

**Table 2:** Language Exposure Characteristics for English-dominant infants in Study 1.

Characteristic	Girls N = 15	Boys N = 10	Total N = 25
	Number (proportion) of participants		
Source of minimal second language exposure			
Spanish	9 (.6)	7 (.7)	16 (.64)
Korean	0	1 (.1)	1 (.04)
French	1 (.07)	0	1 (.04)
Filipino	1 (.07)	0	1 (.04)
Mandarin	1 (.07)	0	1 (.04)
Italian	1 (.07)	0	1 (.04)
Lao	1 (.07)	0	1 (.04)
Estonian	1 (.07)	0	1 (.04)
American Sign Language	0	1 (.1)	1 (.04)
Cantonese	0	1 (.1)	1 (.04)
Relative second language exposure in percent			
1–5	9 (.6)	4 (.4)	14 (.56)
6–10	1 (.07)	1 (.1)	2 (.08)
11–15	4 (.27)	3 (.3)	7 (.28)
16–20	1 (.07)	2 (.2)	3 (.12)
Number of speakers that direct more than one language to the child			
0	7 (.47)	6 (.6)	13 (.52)
1	4 (.27)	2 (.2)	6 (.24)
2	4 (.27)	1 (.1)	5 (.2)
5	0	1 (.1)	1 (.04)
Primary source of second language input			
Mother	7 (.47)	5 (.5)	12 (.48)
Father	2 (.13)	0	2 (.08)
Outside-the-home care	3 (.2)	3 (.3)	6 (.24)
Grandmother	0	2 (.2)	2 (.08)
Family friend	3 (.2)	0	3 (.12)

**Table 3:** MCDI and CCT descriptives for Study 1.

Measure	MCDI comprehension	MCDI production	CCT comprehension
		*M (SD)*	
SES (maternal education)			
4 years of college or more	181.12 (76.42)	49.49 (57.9)	13.3 (7.9)
Less than 4 years of college	182.97 (72.71)	27.48 (21.73)	9.17 (6.76)
Language exposure			
100% exposure to English	195.85 (76.55)	50.04 (56.31)	13.26 (7.91)
80–99% exposure to English	155.56 (63.66)	22.92 (13.69)	8.6 (6.32)
Total	181.86 (74.43)	40.63 (47.82)	11.64 (7.68)

**Table 4:** Distribution of Selected Demographic Characteristics of infants in Study 2 (age in months).

	Spanish monolingual		Spanish dominant	Total
Characteristic	U. S . mean age: 17;5 N = 16	Mexico mean age: 16;21 N = 35	mean age: 16;23 N = 36	N = 87
		Number (proportion) of participants		
Sex				
Female	10 (.63)	12 (.34)	17 (.47)	39 (.45)
Male	6 (.38)	23 (.66)	19 (.53)	48 (.55)
Maternal education				
Below High School	3 (.19)	2 (.06)	10 (.28)	15 (.17)
High School	6 (.38)	4 (.11)	12 (.33)	22 (.25)
Some College	2 (.13)	15 (.43)	2 (.06)	19 (.22)
College Graduate	5 (.31)	10 (.29)	6 (.17)	21 (.24)
Post-Baccalaureate	0	4 (.11)	5 (.14)	9 (.1)
Declined to state	0	0	1 (.3)	1 (.01)
Approximate income^1^				
Less than 34,000	9 (.56)		18 (.5)	27 (.31)
35,000 – 49,000	2 (.13)		5 (.14)	7 (.08)
50,000 – 74,000	1 (.06)		4 (.11)	5 (.06)
75,000 – 99,000	1 (.06)		3 (.08)	4 (.05)
100,000 – 150,000	0		1 (.03)	1 (.01)
>150,000	1 (.06)		0	1 (.01)
Declined to state	2 (.13)		5 (.14)	7 (.08)
Ethnicity				
Hispanic	16 (1.00)	35 (1)	35 (.97)	51 (.99)
White/not Hispanic	0	0	1 (.03)	1 (.01)
Number of conversational speakers				
1–2	0	4 (.11)	1 (.28)	5 (.06)
3–4	7 (.44)	17 (.49)	14 (.39)	38 (.44)
5–6	5 (.31)	4 (.11)	14 (.39)	23 (.26)
7–8	4 (.25)	10 (.29)	5 (.14)	19 (.22)
9–10	0	0	2 (.06)	2 (.02)
Outside-the-home care				
Yes	10 (.63)	3 (.08)	26 (.72)	39 (.45)
No	6 (.38)	32 (.92)	8 (.22)	46 (.53)
Declined to state	0	0	2 (.06)	2 (.02)

**Table 5:** Language Exposure Characteristics for Spanish-dominant infants in Study 2

Characteristic	Girls N = 17	Boys N = 19	Total N = 36
	Number (proportion) of participants		
Source of minimal second language exposure			
English	17 (1)	19 (1)	36 (1)
Relative second language exposure in percent			
1–5	4 (.24)	2 (.11)	6 (.17)
6–10	4 (.24)	8 (.42)	12 (.33)
11–15	1 (.05)	0	1 (.03)
16–20	6 (.35)	7 (.37)	13 (.36)
21–30	2 (.12)	2 (.11)	4 (.11)
Number of speakers that direct more than one language to the child			
0	8 (.47)	6 (.32)	14 (.38)
1	4 (.24)	4 (.21)	8 (.22)
2	4 (.24)	4 (.21)	8 (.22)
3	0	3 (.16)	3 (.08)
4	0	0	3 (.08)
5	1 (.06)	0	1 (.03)
6	0	1 (.05)	1 (.03)
7	0	1 (.05)	1 (.03)
Primary source of second language input			
Mother	3 (.18)	4 (.21)	7 (.19)
Father	5 (.29)	3 (.16)	9 (.25)
Brother/Sister	6 (.35)	6 (.32)	12 (.33)
Outside-the-home care	0	3 (.16)	3 (.08)
Family member/friend	3 (.18)	3 (.16)	6 (.17)

**Table 6:** IDHC and CCT descriptives for Study 2.

Measure	IDHC comprehension	IDHC production	CCT comprehension
		*M (SD)*	
SES (maternal education)			
4 years of college or more	156.50 (67.82)	20.30 (35.98)	8.90 (5.00)
Less than 4 years of college	185.19 (100.01)	42.67 (63.20)	9.36 (6.52)
Language exposure			
100% exposure to Spanish	192.4 (84.1)	42.78 (66.57)	10.04 (6.82)
80–99% exposure to Spanish	148.25 (93.82)	24.47 (36.54)	8.03 (4.48)
Total	173.92 (90.45)	35.12 (56.45)	9.2 (6.01)

## Footnotes

1 Income data were not collected for the Mexico sample as such reports were considered culturally inappropriate.

## Appendix A

**Table:** lexical items, screen orientation, targets by form, and difficulty level for English CCT

Orientation		Target		
Left	Right	Form 1	Form 2	Difficulty level
Dog	Bird	Dog	Bird	Moderate
Sliding	Running	Running	Sliding	Moderate
Mouth	Eye	Mouth	Eye	Easy
Sheep	Lion	Sheep	Lion	Difficult
Orange	Green	Green	Orange	Difficult
Kissing	Hugging	Hugging	Kissing	Easy
Pulling	Swimming	Pulling	Swimming	Difficult
Telephone	Keys	Telephone	Keys	Easy
Kicking	Drawing	Drawing	Kicking	Difficult
Bus	Fire truck	Bus	Fire truck	Difficult
Nose	Foot	Foot	Nose	Easy
Happy	Sad	Happy	Sad	Difficult
Button	Hat	Button	Hat	Moderate
Juice	Banana	Banana	Juice	Easy
Old	New	Old	New	Difficult
Toothbrush	Spoon	Toothbrush	Spoon	Easy
Drinking	Dancing	Dancing	Drinking	Easy
Swinging	Jumping	Jumping	Swinging	Moderate
Horse	Cow	Horse	Cow	Moderate
Milk	Cookies	Cookies	Milk	Easy
Table	Chair	Table	Chair	Moderate
Little	Big	Big	Little	Difficult
Eating	Throwing	Eating	Throwing	Easy
Scissors	Money	Scissors	Money	Difficult
Red	Blue	Blue	Red	Difficult
Truck	Airplane	Airplane	Truck	Moderate
Full	Empty	Full	Empty	Difficult
Bicycle	Train	Train	Bicycle	Moderate
Penguin	Giraffe	Penguin	Giraffe	Difficult
Cheese	Apple	Apple	Cheese	Easy
Smiling	Crying	Smiling	Crying	Moderate
Playing	Sleeping	Playing	Sleeping	Moderate
Bottle	Ball	Ball	Bottle	Easy
Reading	Washing	Reading	Washing	Moderate
Bubbles	Doll	Bubbles	Doll	Moderate
Turtle	Butterfly	Butterfly	Turtle	Difficult
Touching	Riding	Touching	Riding	Moderate
Dirty	Clean	Clean	Dirty	Moderate
Cat	Duck	Duck	Cat	Easy
Pig	Fish	Pig	Fish	Moderate
Girl	Boy	Boy	Girl	Difficult

## Appendix B

**Table:** lexical items, screen orientation, targets by form, and difficulty level for Spanish CCT

Orientation		Target		
Left	Right	Form 1	Form 2	Difficulty Level
Osito	Gato	Gato	Osito	Moderate
Reloj	Tijeras	Reloj	Tijeras	Difficult
Viejo	Nuevo	Nuevo	Viejo	Difficult
Tortilla	Plátano	Plátano	Tortilla	Moderate
Cuna	Basura	Cuna	Basura	Easy
Pelo	Ojo	Ojo	Pelo	Easy
Lavando	Sacando	Sacando	Lavando	Moderate
Cepillo	Plato	Cepillo	Plato	Easy
Secándose	Leyendo	Secándose	Leyendo	Difficult
Leche	Galletas	Galletas	Leche	Easy
Metiéndose	Empujando	Metiéndose	Empujando	Moderate
Cocina	Baño	Baño	Cocina	Easy
Bailando	Caminando	Bailando	Caminando	Easy
Pato	Gallina	Gallina	Pato	Moderate
Llena	Vacía	Llena	Vacía	Difficult
Rompiendo	Tirando	Rompiendo	Tirando	Moderate
Mesa	Puerta	Puerta	Mesa	Easy
Rojo	Verde	Verde	Rojo	Difficult
Cabeza	Mano	Cabeza	Mano	Easy
Borrego	Rana	Rana	Borrego	Difficult
Cantando	Llorando	Llorando	Cantando	Easy
Araña	Mosca	Araña	Mosca	Moderate
Durmiendo	Brincando	Durmiendo	Brincando	Easy
Amarillo	Azul	Azul	Amarillo	Difficult
Contenta	Enojada	Enojada	Contenta	Difficult
Suave	Roto	Suave	Roto	Difficult
Mordiendo	Soplando	Mordiendo	Soplando	Moderate
Naranja	Chile	Chile	Naranja	Moderate
Paleta	Manzana	Paleta	Manzana	Moderate
Enseñando	Apurándose	Enseñando	Apurándose	Difficult
Calcetín	Globos	Globos	Calcetín	Easy
Televisión	Teléfono	Televisión	Teléfono	Easy
León	Oso	Oso	León	Difficult
Jirafa	Pingüino	Pingüino	Jirafa	Difficult
Desayunando	Pintando	Desayunando	Pintando	Moderate
Grande	Chica	Grande	Chica	Difficult
Playa	Campo	Campo	Playa	Difficult
Cuchara	Llaves	Llaves	Cuchara	Easy
Tocando	Tomando	Tocando	Tomando	Moderate
Botas	Falda	Botas	Falda	Difficult
